# The creation of a medical research council in Nigeria: processes, outcomes and lessons

**DOI:** 10.11604/pamj.2024.49.62.41205

**Published:** 2024-10-30

**Authors:** Adesola Zaidat Musa, Ifeoma Eugenia Idigbe, Abideen Salako, Adeleye Hakeem Osho, Olalekan Moses Olayemi, Nnamdi Emmanuel Udu, Oliver Chukwujekwu Ezechi, Babatunde Lawal Salako, Ademola Johnson Ajuwon

**Affiliations:** 1 Nigerian Institute of Medical Research, Yaba, Lagos, Nigeria; 2 Department of Health Promotion and Education, University of Ibadan, Ibadan, Nigeria

**Keywords:** Medical research council, health research, funding, Nigeria

## Abstract

The Nigerian Institute of Medical Research (NIMR) was established by the National Science Technology (NST) Act of 1977 to conduct research on diseases of public health importance, train health professionals, and disseminate research findings. Since its inception, NIMR has successfully performed these mandates. However, the NST Act recommends that NIMR can conduct research, but it did not mandate the agency to fund research. This constraint has limited NIMR's ability to play a proactive active role in competitive funding and conduct of research in response to Nigeria's changing disease profile and other emerging health challenges that have occurred in the country in the last two decades. It is necessary to reposition NIMR to become a Medical Research Council with improved funding to make it fit for purpose like the medical research councils in the Gambia, South Africa, United Kingdom, and United States of America. To achieve this goal, NIMR collaborated with relevant stakeholders including legislators from the Nigerian Senate and the House of Representatives to successfully create and pass a bill to reposition NIMR into the Nigerian Medical Research Council (NMRC). The factors that contributed to the success of this initiative are the recognition by NIMR staff that it is easier to upgrade NIMR to be a medical research council than to establish a completely new agency, the mobilization of representatives of interest groups including staff from national governmental and international non-governmental organizations, health professional associations, academia, and the media. This article describes the process and outcomes of the interventions that led to the creation of NMRC. Countries planning to establish a similar council will benefit from the initiatives in Nigeria by applying the strategies adopted to implement this initiative in their countries.

## Perspectives

Research is essential for the development of any country. Research is particularly relevant in health as it is through research that many breakthroughs have been achieved in the prevention of diseases, development of diagnostic procedures, exploration of innovative treatment, and improvement in effective prophylactic interventions. Health research funding should be prioritized in Africa because this region bears a disproportionately high burden of globally significant diseases. Although Africa comprises 15% of the world's population, it bears 25% of the global disease burden [1,2]. Funding is critical for health research because quality research requires substantial financial investment. However, research can also contribute to prosperity through active collaboration with industry and the development of intellectual properties and patents. Unfortunately, many African countries have low investment in research and development, at an average of 0.4% of the Gross Domestic Product (GDP) [3]. Consequently, the continent contributes only 2% of global knowledge [2]. Other contributory factors to poor research outputs on the continent are the dearth of highly trained scientists, weak infrastructure such as laboratories, talent drain, and lack of political will by African leaders [1,2].

In Nigeria, investment in health research is relevant because of the country's heavy burden of diseases and the persistence of poor health indicators; at 54 years Nigeria has one of the lowest life expectancies in Africa; abysmal childhood and maternal health indices [4]. For example, according to the latest (2018) demographic health survey, only 31% of children aged 12-23 months were fully immunized, 24% of pregnant women did not access any of the antenatal services before delivery, and 31% delivered at home [4]. Well-funded implementation research is needed to address these challenges. Yet, most researchers agree that Nigeria's health research is poorly funded [5-8]. To change Nigeria's health narrative and fast-track attainment of the health-related Sustainable Development Goals (SDGs) the country must increase its investment in health and development from its current level of 0.2% of its GDP [2] to at least 1% recommended by the African Union [9].

Nigeria urgently needs new pipelines for funding health research to address critical areas of need. The establishment of a specialized agency such as a medical research council is one of the ways of achieving this objective. The primary purpose of a medical research council is the coordination and funding of research [10]. Established by parliament in 1913, the Medical Research Council (MRC) of the United Kingdom (UK) is one of the oldest in the world. The creation of a similar council in Nigeria will make significant contributions to improving not only health research funding but also the overall health of Nigerians. Justifying the need for such an agency, the Lancet Nigeria Commission of 2022, consisting of some of Nigeria's foremost health scholars at home and in the diaspora, recommends the creation of a 'National Medical Research Council with 2% of (the) health budget and central government funding to award competitive peer review grants' as part of the required intervention that will transform health policy and achieve universal health coverage and better health for all Nigerians [5]. When established, the Nigerian Medical Research Council will be modeled after existing ones such as the South African Medical Research Council (SAMRC), the Medical Research Council (MRC) in the Gambia, the UK MRC, and the National Institutes of Health in the United States. As an agency of the government with yearly allocation by the parliament, the Medical Research Council uses research, development, and technology transfer to promote the improvement of the health and quality of lives of citizens [11].

In Nigeria, the government agency with a similar status to a medical research council is the Nigerian Institute of Medical Research (NIMR) located in Lagos, Southwest, Nigeria. NIMR was established through the National Science Technology Act (NST Act) of 1977 to conduct research on diseases of public health importance, provide facilities for training of health professionals, and disseminate research findings [12]. NIMR has been delivering these mandates despite the paucity of funding. For example, in addition to its numerous contributions to health research in the country, NIMR recently developed DNA/RNA extraction kit, Monkeypox diagnostic kit, Lassa fever RT PCR test kit, SARS- Cov-2 detection Assay, and the first to sequence COVID-19 index case in Nigeria/Africa. In 2022 alone, NIMR conducted more than eighteen original research on different themes including entomology surveillance, the COVID-19 vaccine, and the discovery of Anopheles stephensis and its sequencing with the bulk of the funding for the studies from external donors. The institute has also achieved its mandate through recent North-South collaborations with reputable institutions of research and health including the US Center for Disease Prevention and Control, the US National Institutes of Health, Wellcome Trust, Novartis, Belmont Foundation, Emory University Atlanta, USA, North-western University USA, New York University, University College London, and University of Cambridge, UK. NIMR's in-country collaborating agencies are the Nigeria Centre for Disease Control, the National Food and Drug Administration, the National Institute of Pharmaceutical Research and Development, and state governments of Abia, Akwa Ibom, Delta, Edo, Kebbi, Jigawa, Ondo, Osun, and Sokoto. However, the emergence of new health challenges coupled with limitations of the law creating NIMR requires that it should be repositioned to a Medical Research Council with a similar mandate to other MRCs worldwide. This upgrade implies that NIMR will have additional responsibilities that will enable it to meet the health needs of the Nigerian population.

In 2022, NIMR, in partnership with the Bill and Melinda Gate Foundation (BMGF), launched a project titled ‘Repositioning the Nigerian Institute of Medical Research to a Medical Research Council’. Twelve months after the launch of the project, both the Senate and the House of Representatives of the Nigerian National Assembly passed the bill for the creation of the Nigerian Medical Research Council (NMRC) and sent it to the President for assent. In this article, we describe the need to reposition NIMR to become NMRC, as well as the processes and strategies adopted to achieve the passage of the bill. The processes and lessons learned from this initiative will be useful for other countries planning to establish a medical research council or other similar projects.

**Need to Reposition NIMR to be NMRC:** the NST Act of 1977 has limited the agency's ability to undertake additional responsibilities needed to appropriately respond to the changing health landscape in the country. The current structure of NIMR is no longer fit for purpose. The need to reposition NIMR to become NMRC is justified from four perspectives. One, the 1977 Act recommends that NIMR can conduct research, but it does not mandate the agency to fund research. Yet, Nigeria's chronic health research funding shortage [5-8] underscores the need for NIMR to competitively fund research. The contribution of NIMR in this regard is expected to complement, not replace, existing government funding mechanisms like the Tertiary Education Trust Fund (TETFund). Access to such funding is also desirable because it will motivate researchers to focus investigations on the country's priority health problems and become less dependent on foreign donor funding, which is typically short-term and intended to promote the interests of the donors.

Two, many significant changes have occurred in the disease profile in Nigeria in the last two decades that require that NIMR take on additional responsibilities of funding emerging health challenges in the country. For example, Nigeria is experiencing an epidemic transition where Non-Communicable Diseases (NCDs) are now major killers along with infectious diseases [13]. According to the latest NCD progress report, NCD-related deaths now account for 29% of all deaths in Nigeria [14]. The major NCD-related causes of mortality are cardiovascular disease, cancers, chronic respiratory diseases, and diabetes. The associated risk factors are harmful use of alcohol, physical inactivity, salt/sodium intake, tobacco use, raised blood pressure, and obesity [14]. This situation calls for more innovative interventions through research to address risk factors and reduce deaths associated with NCD. When created, NMRC will be able to lead and fund research not only on NCD but also on other emerging diseases of public health importance in the country.

Three, the creation of NMRC and the improvement in funding that will follow will contribute to the attainment of the health-related SDGs in Nigeria and the global community. All hands should be on deck to achieve this objective because Nigeria could not achieve any of the health-related Millennium Development Goals (MDGs) and is only making modest progress towards attaining the health-related SDGs [5]. Of relevance is SDG 3, which is to ensure healthy lives and promote well-being for all at all ages [15]. Some of the key targets for this SDG3 are the reduction in maternal, and child deaths, and prevention of premature deaths from NCDs. The transitioning of NIMR into NMRC will contribute to the attainment of these targets because of the better funding of research that can be achieved through intra-mural or extra-mural competitive funding mechanisms.

Finally, the experiences of the successes recorded by Medical Research Councils in South Africa, the Gambia, the United Kingdom, and the USA suggest that the creation of NMRC will serve as a catalyst that will transform the health research landscape from one characterized by limited funding, low research output to one with sufficient funding and high research outputs. Nigeria should learn and leverage these models of MRCs that work.

**Figure 1:**
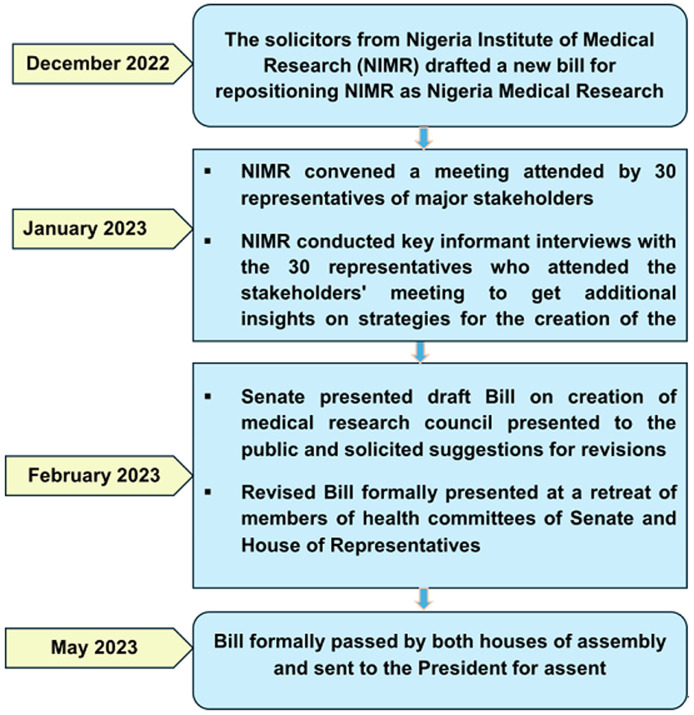
flow chart of activities implemented in the passage of the bill for the creation of the Nigerian Medical Research Council

The processes, opportunities, challenges and solutions: NIMR and its partners took advantage of existing opportunities and analyzed the challenges to derive solutions in the process of implementing three activities which culminated in the passage of the bill to reposition NIMR to become NMRC (Table 1). These include convening a stakeholders meeting, in-depth interviews of stakeholders, and a one-day retreat with members of the National Assembly. A summary of key activities is described in (Figure 1).

**Table 1:** opportunities, challenges, and solutions to the creation of a medical research council in Nigeria

Opportunities	Challenges	Solutions
NIMR has an existing structure to function as a medical research council. NIMR has adequate manpower to perform the role of a medical research council. NIMR has delivered on its mandate as an agency of government responsible for conducting health research .	Health-related research is under-funded in Nigeria. The growing burden of both infectious and non-communicable diseases in Nigeria requires new channels of funding. The law that established NIMR does not allow it to directly fund research. Some stakeholders may be against the creation of a new agency .	The proposed Medical Research Council will serve as an additional agency through which health research can be funded. NIMR has the expertise to conduct research to address infectious and non-communicable diseases. It is easier to amend the law creating NIMR than to create new ones. All stakeholders were mobilized and involved in the process of creating a medical research council.

**The Stakeholders meeting:** the stakeholders meeting was held in Reiz Continental Hotel in Abuja, Nigeria's capital city, between January 25-26, 2023. Thirty (30) professionals drawn from government agencies, both houses of the National Assembly (NASS), international non-governmental organizations, professional associations, academia, representatives of Medical Research Councils from the Gambia, UK, and South Africa, and the media, attended the meeting. The objectives of the meeting were to present to the stakeholders the plan for repositioning NIMR to become NMRC, review the draft bill for creating a medical research council in Nigeria, and solicit ideas and suggestions for their implementation. The staff of NIMR presented the existing structure, functions, and achievements of NIMR and highlighted the limitations of the NST Act of 1977 especially as it relates to funding of research. The representatives from the MRC Gambia, SMRC, and UK MRC also made presentations on the structure, functions, and achievements of these agencies.

The objectives of the stakeholder's meeting were achieved in the sense that participants discussed, suggested revisions to the bill, and provided strategies that will successfully reposition NIMR to NMRC. Among the essential suggestions was the replacement of the designation of the Chief Executive Officer of NMRC from President to Director General in line with the nomenclature for the administrative heads of other government agencies in the country. Stakeholders also suggested that the council's primary mission should be to fund, coordinate, and carry out health research. Another important outcome of the meeting was that representatives of the relevant agencies including the Federal Ministry of Health, the NASS, the World Health Organization, the Postgraduate Medical College, and the Nigeria Academy of Science, made commitments to provide technical assistance and support required to ensure that the NASS pass the bill when it is presented.

**Interviews with stakeholders:** the interviews were conducted after the stakeholder's meeting. The goal of the interview was to provide a detailed situational and landscaping analysis of stakeholders' perceptions, opinions, and experiences on the need to establish an NMRC. It was also done to explore if establishing an NMRC was acceptable, feasible, and sustainable and to identify the facilitators and potential barriers to achieving this objective.

Thirty (30) professionals working in research and academic institutions were interviewed. Half (15) of the respondents consisted of those who attended the stakeholder's meeting while the remaining 15 were health researchers and scholars with 10-40 years of professional experience living in and outside Nigeria including the Gambia and the USA. The criteria for the selection of those interviewed during the stakeholders meeting was that they attended that meeting. The criteria for the choice of other respondents were that they had a previous research collaboration with NIMR or had some affiliations with an MRC or other international research institutes. Face-to-face interviews were conducted with respondents who were available during the stakeholders' meeting. Telephone interviews or virtual meetings were held on Zoom for others at a date and time that was convenient for them. All interviews were recorded on digital audio tapes after respondents provided informed consent; each interview lasted between 20-40 minutes.

The key findings from the interviews were that many participants believed that there is a need for Nigeria to have an MRC. Some participants stated that the creation of a medical research council is feasible because the NIMR was initially called the Nigerian Medical Research Council and has similar mandates. Most interviewees stated that establishing and running the NMRC will be sustainable if the right structure, human resources, and appropriate funds are provided. Respondents believe that funding is pertinent in ensuring the proper establishment, smooth transition, and effective implementation of an MRC. They suggested that funding should be sourced from the Federal Government, the organization of extramural programs, international donor organizations, and buy-in from captains of industries and non-governmental organizations. They also stated that the NMRC could be sustained if positive strategies were implemented to generate income to run the institution. Some of the facilitators identified include the ability of the management of NIMR to successfully mobilize all stakeholders including researchers, academicians, politicians, and captains of industries to support the plan. Other suggestions provided for the successful creation of the NMRC are reflected in the verbatim quotations below:

*“The council will stand and, it is good to do very strong advocacy, as we call it evangelism. Research evangelism must get to the highest quarters, from the Presidency, down to the National Assembly down to the Ministers and even to the Local Government Chair”. (S20, Female, 56 years)*

*“Engaging other stakeholders and promoting advocacy tools to provide great information about the MRC and then also developing programs to show the value, communicating the value of having an MRC to the general community.” (S22, Female, 65 years)*

**Retreat of the NASS:** the draft bill was presented to the public at the NASS complex in Abuja on Tuesday, January 31, 2023. Following this process, NIMR in collaboration with the representatives of the NASS organized a one-day retreat with members of the Senate Committee on Health/House Committee on Health Services on February 13, 2023, at Transcorp Hotel, Abuja. The attendees deliberated on all the suggestions received during the public hearing. The key agreements from the meeting were that the title of the Chief Executive of the Council should be ‘Director General', that members of the Board of the Council should include the Chairman of the National Health Research Ethics Committee, and that the annual budgetary allocations from annual appropriations of the Federal Government should be provided to the council. After revisions were made and following the presentation and discussion of the bill by the Senate, the Nigerian Senate formally passed the Bill on February 13, 2023. The House of Representatives ratified and passed the bill on May 23, 2023. The bill has been sent to the President for his assent.

**Lessons Learnt:** the timeframe of approximately one year it has taken to develop a bill and have it passed by the NASS is remarkable considering the elaborate processes involved in creating legislation in Nigeria [16]. Four lessons can be identified from the processes involved in repositioning NIMR as NMRC. First, it was feasible to reposition NIMR to be NMRC because NIMR and its partners adopted a practical approach to the task namely that it is easier to upgrade NIMR to be a council than to create an entirely new agency. Since NIMR is the agency of government responsible for coordinating medical research in Nigeria, it is logical to increase the functions of the organization to make it more efficient and fit for purpose. The repositioning of NIMR will enable the utilization of existing robust medical and bio-medical research infrastructure including Nigerian medical scientists to promote the improvement of health in Nigeria. The lesson here is that upgrading an existing agency performing a similar function is more straightforward than creating a new one.

Second, the mobilization and involvement of all relevant stakeholders are essential to the initiative's success. The composition of the stakeholders who attended the meeting or participated in interviews was not only diverse but also inclusive. The involvement of professionals representing all relevant sectors including government agencies, international organizations, local regulating agencies, and the media resulted in robust discussion and valuable suggestions. The participation of two serving members representing both houses of the NASS provided insights into the operations of the assembly, and the identification and revision of potential controversial clauses in the bill. The meeting also created an opportunity for NIMR staff to clarify the structure and functions of the proposed NMRC. It was clarified for example that the proposed council is not a regulating agency for research, instead its core functions are to fund, coordinate, and conduct medical research in Nigeria. The presence of all key stakeholders at the meeting forestalled the risk of objection from interest groups, a common problem of bills developed without input and support from major interest groups [17]. The lesson is the need to ensure that all agencies and representatives of all relevant stakeholders are involved in the process of creating similar agencies.

Third, the presentations of representatives from Medical Research Councils in the Gambia, South Africa, and the UK, served as an eye-opener to the potential benefits of creating a similar council in Nigeria. The management of these councils and their impact on the overall health of citizens of the respective countries were also useful and important. Some of the key points from the presentations were: that sustainable funding for the council should be provided by the legislature, that the council should be scientifically independent, and that the council should generate funds through active collaborations with industries, and the development of intellectual properties and patents. The lesson is that new organizations can derive inspiration from those successfully running and managing similar agencies.

## Conclusion

Since its inception, NIMR has been successful in delivering on its mandate. However, the NST Act that established the agency has limited its ability to play a more active role in funding and conducting research in response to the changing disease profile and other health challenges that have occurred in Nigeria during the last two decades. There is therefore the need to reposition NIMR to NMRC with better funding to make it fit for purpose like similar councils in the Gambia, UK, and South Africa. NIMR in collaboration with BMGF worked with stakeholders including representatives of both houses of the NASS to ensure the passage of the bill. African countries planning to establish similar medical councils can benefit from Nigeria on the processes involved in the implementation of this initiative. This has been made possible because of the strategies adopted including involvement of an inclusive group of stakeholders and contributions of representatives of existing medical research councils from African countries and the UK.
